# Behavioral Acoustic Emanations: Attack and Verification of PIN Entry Using Keypress Sounds

**DOI:** 10.3390/s20113015

**Published:** 2020-05-26

**Authors:** Sourav Panda, Yuanzhen Liu, Gerhard Petrus Hancke, Umair Mujtaba Qureshi

**Affiliations:** 1 Department of Computer Science and Engineering, University of California, Riverside, CA 92521, USA; spand009@ucr.edu; 2 Department of Computer Science, City University of Hong Kong, Hong Kong, China; yuanzhliu3-c@my.cityu.edu.hk (Y.L.); umqureshi2-c@my.cityu.edu.hk (U.M.Q.); 3 Department of Telecommunication Engineering, Mehran University of Engineering and Technology, Jamshoro 76062, Sindh, Pakistan

**Keywords:** side-channel attack, personal identification number, biometric verification, PIN entry device

## Abstract

This paper explores the security vulnerability of Personal Identification Number (PIN) or numeric passwords. Entry Device (PEDs) that use small strings of data (PINs, keys or passwords) as means of verifying the legitimacy of a user. Today, PEDs are commonly used by personnel in different industrial and consumer electronic applications, such as entry at security checkpoints, ATMs and customer kiosks, etc. In this paper, we propose a side-channel attack on a 4–6 digit random PIN key, and a PIN key user verification method. The intervals between two keystrokes are extracted from the acoustic emanation and used as features to train machine-learning models. The attack model has a 60% chance to recover the PIN key. The verification model has an 88% accuracy on identifying the user. Our attack methods can perform key recovery by using the acoustic side-channel at low cost. As a countermeasure, our verification method can improve the security of PIN entry devices.

## 1. Introduction

**Figure 1:**
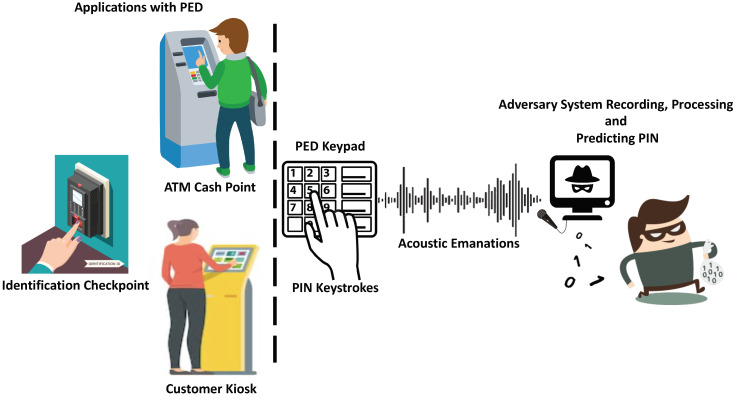
PIN Attack: Acoustic emanations generated from PED keystrokes are recorded and processed to predict a PIN key by an adversary.

The Internet of Things (IoT) refers to a network of tiny small wireless sensors that communicate with each other via the Internet [1]. Today, IoT provides a wide range of consumer applications [2]. IoT devices are resource constrained devices which makes them an attractive target for attacks [3]. To access different applications and services, user authentication and verification is the first layer of security. Failing to authentication and verification process leads to denial of service. For example, authentication and verification processes are commonly used in Automated Teller Machines (ATMs) and Point of Sale (POS) terminals which are resource constrained systems. The process of authentication and verification is important as it allows legitimate personnel to enter and perform various operations in their respective environments. Personal Identification Number (PIN) keys or numeric passwords are widely used in such resource constrained environments. PIN key is basically small and unique string of data that is ubiquitously used for user authentication and verification. Usually, PIN key is typed into PIN entry device (PED) or terminal as shown in Figure 1.

This paper focuses on exploring the security vulnerability of such PED terminals. Passwords are one of the most important personal authenticating methods in the world. Substantial research has been done on password authentication, including the reasons how users set their passwords, the rules that users use to set passwords and the methods to infer passwords [4,5,6,7,8,9]. When space or cost is limited, for example, in the case of POS terminals or ATM machines, it is preferable to use numeric passwords, in other words PIN keys.

The Payment Card Industry (PCI) Standard Council defined the standards of security and testing requirements for certification of devices used for PIN entry in payment and transaction for the first time in 2002 [10]. One of the most significant requirements for PEDs physical security is that there should not be a feasible way to infer the entered digits on PEDs by recording and analyzing the sound, electromagnetic emissions, power consumption or other external information [10]. Theoretically, it should not be possible to perform an attack on the PEDs. However, whether the standards are strictly implemented is an open question. Several security problems of PEDs not mentioned in the certification already exist. Example are tampering and PIN/card details logging [11], card wedge allowing transaction approval with no PIN [12], tampering with Point of Sale (POS) terminals to log PIN and payment data, infecting these devices with malware [13], and ineffective random number generation by PEDs for cryptographic functions [14].

This paper aims to analyze the security vulnerability of modern PEDs, for example ATM keypads or POS terminal keypads. ATM keypads allow users to input numeric passwords or PINs. The PIN entry is quick (small instances of time) because it comprises of a short string of data (4–6 digits) and it is only typed once by the user. This makes the information inference extremely challenging and difficult compared to information inference from traditional keyboards in which the user is expected to type for long instances, which increases the inference likelihood of the user data. Therefore, the goal of this paper is to study two aspects of this security issue, i.e., attacks on PEDs and the safeguarding of PEDs against attacks. First, we investigate the possibility of inferring the PIN key. A system that is able to record the sound emissions of keystroke from the PED keypad (for example, ATM Keypads or POS keypads) and extract features which are used to predict the PIN, is shown in Figure 1. Secondly, we look at the feasibility of these acoustic emissions be used in enhancing the security level of PEDs. The assumption is that each user exhibits a unique behavior while entering the PIN keys. This behavior can be seen as a fingerprint and as a way to verify the key holder’s identity, thus acting as an authentication layer that is able to prevent the attack or key theft, and at the same time preserving the public’s confidence in these systems. Thus, the contribution of this paper is as follows:

- An investigation into the security vulnerability of PEDs by performing a PIN key recover attack on the random six-digit PIN number by using the acoustic emanation generated by the PED keypads.
- Proposing the behavioral acoustic emanations as a countermeasure and verification method for a PIN key user’s identity.

The remainder of the paper is organized as follows: Section 2 presents our review of work presented in the literature on PIN attacks and preventative measures. Section 3 presents the key PIN recovery attack, along with the methodology through acoustic emanations.

Section 4 explains the idea of user verification by using behavioral acoustics generated from the PEDs to authenticate user identity and prevent key PIN attacks. Section 5 concludes the paper.

## 2. Literature Review

In this section, we review the most relevant work in the context of information inference of user data (Password, Key or PIN). Generally, users lock their information by typing a password or PIN into the system via a keyboard or keypad. The password or PIN entry into the system generates different kinds of emissions, which are exploited by adversaries to breach the system’s security for information theft purposes. In this section, we first present a review of the literature on emission security, side-channel analysis and key recovery methods from different sensors to have an insight into different attack models and approaches used to recover a key from the system. In the next section we shall review countermeasures and approaches present in the literature to prevent key theft.

### 2.1. Emission Security and Side-Channel Analysis

Early in the 1960s researchers noticed that systems could leak information unintentionally by electromagnetic, optical or acoustic emissions [15,16]. This type of work, including obtaining information from unintentional emission analysis and preventing emission from leaking, is known as TEMPEST [17] in the intelligence and military communities. One feasible method to infer data was intercepting electromagnetic emissions from cables, such as RS-232 or PS/2 [18,19]. Other attacks also exist, for example, recovering printed documents by the sound of the printer [20], or reconstructing the sent data from communication equipment by the LED status indicators [21]. When the display technology was improved, some researchers also aimed at monitoring video displays by both electromagnetic and optical emanations [22,23,24]. The latest research can even eavesdrop from a cell phone screen or from tablet screen [25,26,27,28]. The work presented in [25], the authors presents a side-channel attack to retrieve PIN from a cell phone screen. The attack was devised by using two microphone or mics embedded in a cell phone to listen the digit taps when the PIN is entered. The authors used Time Difference of Arrival (TDOA) technique to infer the location of each sound source and then it maps with Keypad layout to identify the character/digit with accuracy greater than 50%. The limitation of the work is that authors attempted to identify single character/digit each time instead of the combine PIN. The emanations generated during a PIN entry (quick taps) can result in similar sounds which can lead to false positioning of the digits. The work presented in [26], the authors presents a mathematical model used to devise character and digit recovery attack from victim’s cell phone screen. The attack derives prior information such as location of the victim cell phone in relative the location of the adversary and studies. The authors report that to successfully infer the character or digit tap information, the distance between the victim and adversary needs to be less than 60 cm. However, in [27], the authors present acoustic side-channel attack to retrieve the lock patters by using the mic embedded in an android cell phones. The authors use a cellphone application to record the emanations of lock pattern and used to noise rejection filter, signal segmentation, relative movement measurement to infer the pattern of the fingertips. The author reports an accuracy of 72% for successfully recovering screen lock patterns and propose dynamic instead of linear patters to strengthen the security. From these successive attacks on the display, we can draw a conclusion that data reconstruction from emissions is highly correlated with the hardware design of the destination devices. The same applies to keypads. The physical design of keypads may lead to various emissions, such as sound and vibration. Side-channel analysis aims at reconstructing the plaintext, passwords or other information by gaining information from the hardware. In general, the side-channel attack is not aggressive. Instead of a direct attack on the information, it is targeted at gaining data from timing [29], power consumption [30] or electronic emissions [31] while the device is working. Acoustic side-channel attacks are proved to be possible [32]. The side-channel attack is so useful that it even changed the threat model, design and certification testing of secure hardware since the late 1990s [33,34]. From the above cases we can draw the conclusion that when designing a security component, it is necessary to consider side-channel information leakage.

### 2.2. Key Recovery from Sensors

In recent years, smart devices are widely used in the industrial environment. The sensitive information may be unintentionally captured by the devices. If malicious applications were installed in the devices, this may lead to information leakage [35]. In fact, the side-channel attacks on the recovery of passwords by analyzing the data from different sensors, such as images, sound and acceleration, have already been proved to be possible [36,37,38,39,40].

Simon et al. (2013) suggested a new side-channel attack on PINs by gathering data from a mobile phone’s front camera and microphone [41]. They used a gaming approach (game application) to perform the attack, including data collection, feature extraction, training a model, recording data and analyzing results. They used a Support Vector Machine to train the data from two users and got 35–50% prediction accuracy. Next, they tried to recover a PIN key with video and audio streams, but found that this method required 5 or more attempts to achieve an accuracy of more than 50%.

Owusu et al. (2012) suggested that accelerometers can be used to infer passwords [39]. They collected data in two ways. The first was the area mode, dividing the screen into a 10 by 6 button array. This mode was used to test how the sampling rate can affect the accuracy of key inference and the information leakages of different screen regions. 1300 key presses were collected in this mode. The result showed that a sample rate equal to or above 100 Hz can present the best results. The other method was character mode. This mode was used to test the keystroke reconstruction attack. Acceleration data affiliated with key pressing was recorded with pre-processing for feature extraction and used this data for classification. The authors found that a Random Forest classification algorithm delivered the best results. It successfully cracked 59 of 99 passwords. Liu et al. (2015) suggested a side-channel attack on keystrokes by using accelerometer data of a user’s smartwatch [40]. To attack the POS terminal, they recorded the POS terminal keypad motion and used K-Nearest Neighbor to select the best option. The authors recorded 4920 movements, including 3720 motions between two numbers and 1200 “Enter” button movements. The highest accuracy for one-time recovery could reach 65%. Since most POS machines allowed a user to input 3 times, the accuracy could be improved to around 85%.

Asonov et al. (2004) used acoustic emanations and performed the attack to recognize the key pressing by applying a neural network [36]. They extracted features from raw acoustic signals by applying a Fourier Transform with 2–3 ms or 8–10 ms window to the signals to find push peaks and trained the system with user keystrokes on a standard QWERTY keyboard.

The result showed that a network trained for one person can be applied to attack another person’s key pressing on the same keyboard. However, when the keyboard changed, the success rate dropped to 28%. This means that the neural network is only applicable on the keyboard that is used to generate the training set. The main reason for sound differences between keys is the different positions on the keyboard plate. We make a conclusion in this paper that the sound tune was actually used to differentiate between the key pressings.

Zhuang et al. (2005) tried to improve the method. They used cepstrum to extract features. In addition, they used unsupervised key recognition (K-Means) to classify the keystrokes with a larger number of keys of the keyboard [37]. Then they applied a Hidden Markov Model to determine the key sequence to improve the accuracy along with spelling and grammar checking to yield the accuracy rate and trained their classifiers. Their experimental results showed that linear classification and Gaussian mixtures had a better performance than a neural network.

Zhu et al. (2014) presented a method to recover keystrokes (push and release) without context analysis [38]. Their analysis indicated that the minimal distinguishable distance between two sound sources was (343 m/s)/(44.1 kHz) 0.77 cm, which is less than the distance between two adjacent keys on the keyboard. They used two or more mobile phones in different places to record the sound from the keyboard, which were pre-processed to find the sound peak in each 100 ms. The experiments showed that 3 or more phone microphones lead to more accurate results. Time sync was a sensitive problem in this method. To address this problem, they used a fixed distance between keyboard and phones to synchronize the time. The accuracy of this method was quite acceptable, as more than 72.2% keys were recovered.

Cardaioli et al. suggested a PIN inference method by analyzing user’s typing behavior [42]. The behavior features are extracted from audio signal; They used thermal camera to retrieve more information to raise the accuracy. Faria et al. performed a new side-channel attack on PIN pads [43]. They used two inner mics to record audio and differential analysis of the vibration signal differences between the keystrokes to infer the PIN.

### 2.3. Preventative Measures against Key Recovery Approaches

Researchers supposed various methods of attack on PEDs. These attacks focused on the key stroke to recover the PIN. Many countermeasures against side-channel attacks were suggested, including changing the physical architecture of devices [44,45] and adding noise to the side-channel emanation [46,47]. Another possible method is adding an additional verification layer combined with PINs. There are three main streams of verification methods, i.e., biometrics, keystroke dynamics and active authentication.

#### 2.3.1. Biometrics

Biometrics are unique physical traits and behavioral characteristics that work as excellent candidates for automated recognition and authentication [48]. Conventionally, biometrics are sub-classified into Physiological and Behavioral characteristics [49]. Instances of physiological authentication systems include fingerprint scanning, face recognition, and DNA recognition. These are static features and are unique across a large domain of users. On the contrary, behavioral traits include the amplitude and pitch of a user’s voice, the way people sign their names, and their keyboard typing patterns. Unlike the former, behavioral characters are dynamic and are revealing of the user’s psychological composition (cognitive fingerprint) instead of stable physical features [50]. Therefore, resilience is a concern as a sudden and gradual deviation from expected user behavior is inevitable and requires a robust system capable of adapting to change over a prolonged period. In comparison to physiological traits, behavior authentication is inexpensive and passive.

Researchers have also asserted that for the foreseeable future, biometric services will not eliminate issued ID cards and password PINs, but rather complement the identification process and accountability of the authentication workflow [51]. Furthermore, behavioral biometrics never yield an absolute match between the expected and the input during the verification step. As a result, the False Acceptance Rate (FAR) and the False Rejection Rate (FRR) are not as low as the ones provided by physiological verification. Therefore, behavioral factors alone cannot constitute a reliable authentication system for the various extrinsic factors involved, such as mood and fatigue. Ogihara et al. (2006) proposed a verification method using biometric features [52]. By both calculating the similarities of key press timing between current operators and users and comparing extracted hand shape features, they can improve the security of ATM authentication. One method was key press timing. To process a 4-digit PIN, they calculated the press and release time for each key, and the time between two adjacent PIN key number. Another method was extraction of hand shape features. If the two methods were used separately, the error rates were between 10% to 25%. If the two methods were combined, the error rate was reduced to 1.1%.

#### 2.3.2. Active Authentication

Presently, active authentication is the norm when it comes to validating the identity of a user for access applications [53,54]. It requires explicit user interaction (a passphrase or inputting a fingerprint, etc.) which authorizes the user based on the legitimacy of the input. If successfully authorized, the user is provisioned system resources until session timeout, or until the user voluntarily logs out. Any activity during this session period is accounted to the authenticated user and an impostor physically present at the system cannot be detected. Furthermore, after a session timeout, users must digress from their intended objective in mind to perform the cumbersome authorization step, yet again. Active authentication alleviates these complications by continuously authenticating users on the way they interact with the environment. These interactions construct the cognitive fingerprint of the user and reveal how the brain processes the technology in hand. Active authentication has the potential to detect intruders after successful authentication, and cases where the user’s password have been compromised. Additionally, since the interaction depends on the technology in hand, software could also be devised to help employers distinguish between employees working from home and office. This research work argues that the latencies between the successive keystrokes, key duration, finger placement and applied pressure can be used to construct a unique cognitive signature for a user in a non-intrusive manner [51]. This work studied Euclidean distance, non-weighted probability, and weighted probability algorithms to determine the authenticity of a user typing a username and password. The authors report an authentication accuracy of 83.22% with Euclidean distance, 85.63% accuracy with non-weighted probability and 87.18% accuracy with weighted probability.

The works reviewed above explains different PIN recovery attack models used along with countermeasure approaches present in the literature. For PIN recovery attack, our focus is to devise a simple yet effective methodology. The works [13,38] provide us with an interesting insight that motivates the consideration of a PIN key recovery attack on modern PEDs. We derive our methodology from these two works that focus on using time intervals of keystrokes generated from acoustic emanations of the PEDs. The methodology is composed of simple common-off-the-shelf (COTS) devices which consider features that can be easily be extracted from acoustic emanations. PIN Key recovery through acoustic emanations is unique and the methodology is devised such that it act as a general framework that can be readily formed and applied to any modern PED that uses small strings of data. It covers groups of PINs no matter that they are random or not, which is different. This makes our methodology simple yet effective compared to attack models reviewed above. Also, our approach overcomes the shortcomings and limitations of [13,37,43] and the feasibility of application in real life is improved compared to the method of [25,38].

To defend PIN attack and verify the identity of the person when entering a PIN, we used the acoustic emanations to extract the timing feature, which saves save many time, space and power compared to Ogihara’s method [52], which used camera to record the motion of hand. Moreover, microphones are cheaper than cameras. In comparison, our method’s cost is lower, both in the required devices and computational power. Compared to current active authentication [53], the lower cost is also an advantage of our method. Active authentication requires a fingerprint, this means we need a fingerprint sensor, which is more expensive than a microphone, to do the recording. If we use a mobile phone to perform active authentication, we need to send messages to the user. The vendor will charge for the message fee when authentication is performed, whereas our preventative measure eradicates these issues. The proposed behavioral acoustic detection for user verification is simple and novel that can successfully prevent a KEY PIN recovery attack and acts as an authentication security layer for modern PEDs. In the subsequent sections both the PIN recovery attack based on acoustic emanations and verification based on behavioral acoustics of PIN key users are explained.

## 3. Acoustic Side-Channel PIN Recovery

The advent of Artificial Intelligence (AI) and automation is slowly obviating the need for cashiers in supermarkets and grocery stores, making self-checkout lines increasingly popular. Although these services are convenient from the consumer’s outlook, they do pose serious security implications for them. For instance, the checkout kiosk can collect data pertinent to the user’s behavioral characteristics and exploit them to recover data entered over third party trusted interfaces using side-channel analysis. To better understand this, consider the following application-threat model.

### 3.1. Application-Threat Model: PIN Key Recovery by Using Acoustic Emanations at Customer Kiosks

In this application-threat model, we consider an automated self-check-out machine in an industry canteen that does the job of calculating the total bill and collecting the payment using a debit card transaction. The kiosk is assumed to be fitted with a microphone device that is equipped to connect to the customer service for assistance. Now, when the customer completes the item checkout process, he is prompted to enter some information (e.g., a telephone number) or key that allows the microphone to collect data regarding the user characteristics using keystroke acoustic emanations. Once this system is trained with the user’s habitual typing patterns, it prompts the user to enter his debit card and PIN number, which is obfuscated by the trusted banking interface. The idea is to exploit these acoustic emanations carefully such that the system can predict the PIN. To perform such an attack, the methodology followed is explain below.

### 3.2. Methodology

**Figure 2:**
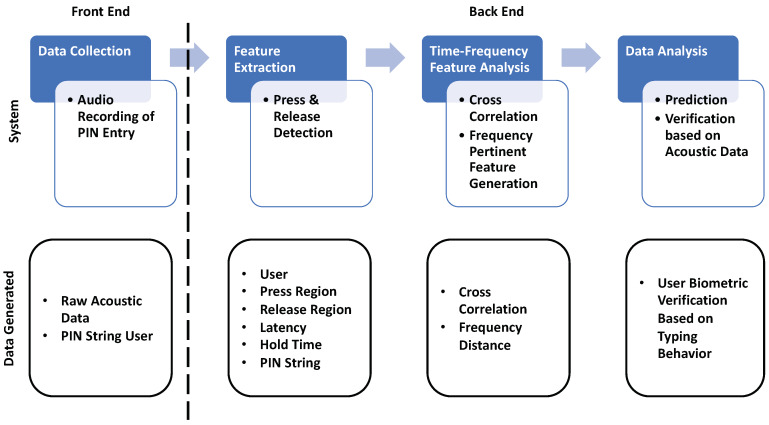
Experimental workflow diagram.

Our methodology consists of four systems that will be elaborated on in the following sections of this paper, namely (a) Data Collection, (b) Feature Extraction, (c) Time-Frequency Analysis, and (d) Acoustic/Data Analytics. The data collection is considered the front end of the system, whereas every other phase resides in the back end as shown in Figure 2.

#### 3.2.1. Data Collection

**Figure 3:**
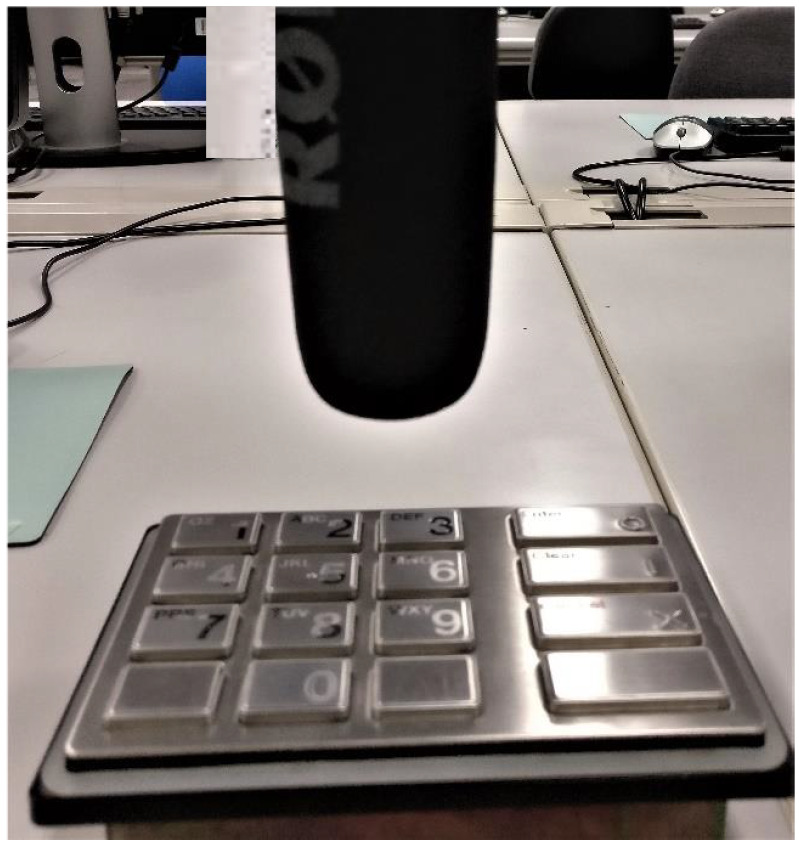
Experimental setup.

With reference to the application-threat model presented above, we are mostly interested in the emissions emitted from the keystrokes during the PIN entry. We carried out our experiments on an ATM PIN Entry Device and recorded the emitted acoustics using a Video mic recorder as shown in Figure 3.

The users were asked to memorize a six-digit numbered PIN and adapt themselves to this new PIN sequence by entering it on the keypad device until familiar with the keystroke patterns. Once the user was trained, we placed the PED under a recording tool (Figure 3) and asked the user to enter the memorized PIN. After finishing the PIN entry, the raw acoustic data is fed to the workstation for future acoustic analysis. Each PIN sequence is entered four consecutive times before proceeding to the next PIN sequence in the list. This list consisted of four PINs and comprises all possible transitions ranging from (a) short, (b) long, and (c) diagonal. From here on, we constructed an audio signal repository of 15 users, with each user entering four distinct PINs for a total of four times. Throughout the experiment, the acoustic data was collected using a Rode microphone designed for typical personal audio recording. The tool collects data from a directed source in the frequency range 40 Hz to 20 kHz, together with an 80 Hz high-pass filter that prevent low-end noise from being recorded. All recordings were triggered by a MATLAB script that instructed the audio recorder to collect data for a certain interval.

#### 3.2.2. Feature Extraction

**Figure 4:**
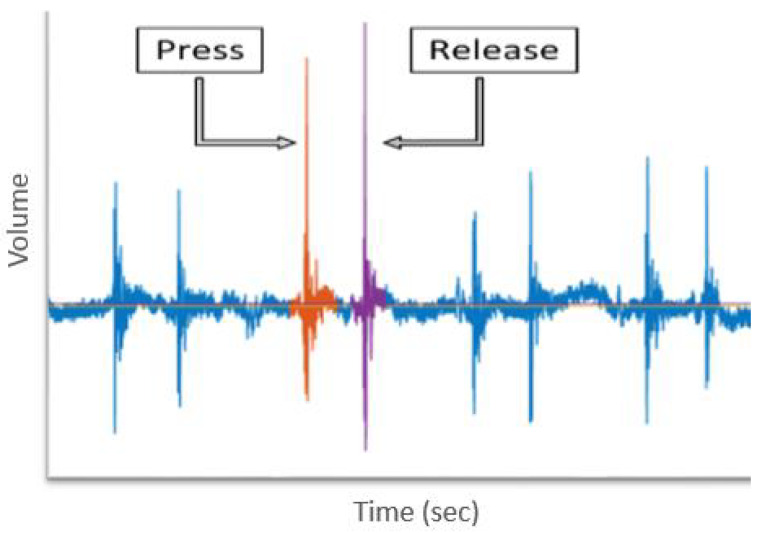
Press and Release region in a keystroke generated acoustic signal.

As explained in [36], regions in the keystroke generated acoustic signal can be associated with particular events, specifically the press and release events as shown in Figure 4. Every analysis in this paper considers these two regions (Press & Release) as separate entities and excludes the intermediate region in-between. Therefore, estimations for distance and similarity metrics are evaluated for corresponding events as proposed in [36,55]. For instance, the pairwise similarity between ‘*f*’ and ‘*g*’ is calculated in the following manner.

(1) $$\begin{array}{c} similarity_{press}=pairwise_{Similarity}\left(f_{press},g_{press}\right) \end{array}$$

(2) $$\begin{array}{c} similarity_{release}=pairwise_{Similarity}\left(f_{release},g_{release}\right) \end{array}$$

(3) $$\begin{array}{c} similarity_{overall}=\frac{1}{2}\left(similarity_{press}+similarity_{release}\right) \end{array}$$

**Figure 5:**
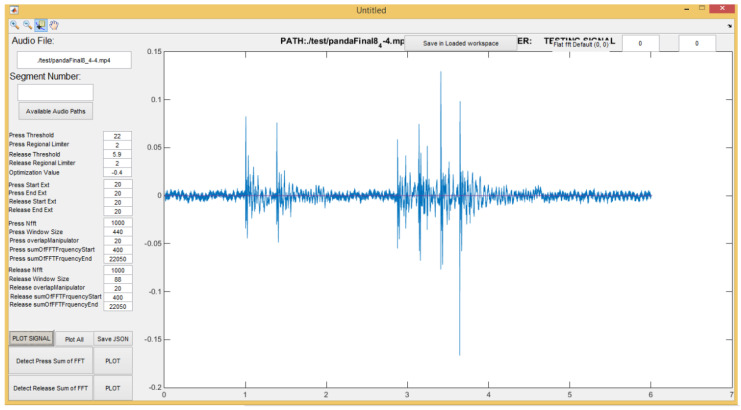
Custom MATLAB feature extraction console.

**Figure 6:**
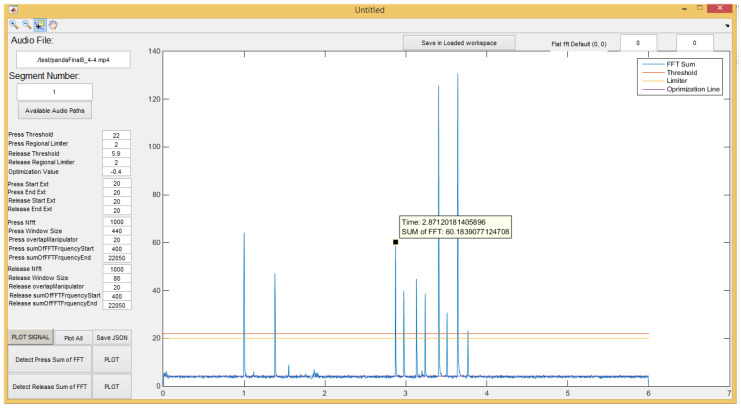
Sum of FFT coefficients to detect Key Pres.

**Figure 7:**
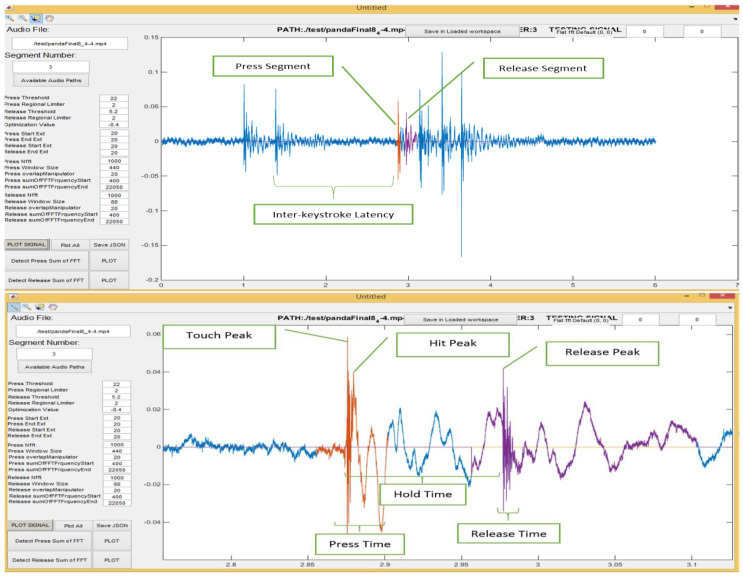
Various features analyzed in this project.

Figure 5 and Figure 6 demonstrate the feature extraction console constructed in MATLAB to generate the feature vector by using a Fast Fourier Transform (FFT) that represents the PIN entry acoustic signals. This console employs key press detection techniques explained in [55] to pinpoint the beginning of each key press and derives the features that are conducive to this research. Most of these features are represented in Figure 7 and Table 1.

To make the machine-learning algorithms work, it is important to employ appropriate signal processing techniques to engineer features that serve as meaningful inputs. Although we later show that Table 1 consists of the reliable features when it comes to user verification, in the next section of this paper we discuss the feature engineering pertinent to time-frequency analysis as a part of our methodology.

#### 3.2.3. Time-Frequency Analysis

This section discusses the cross-correlation analysis that we used along with frequency distancing to predict user PINs solely from the acoustic signals. Unlike other papers, we cannot employ dictionary models or character pair frequency analysis, because the PIN entries are completely random.

1. Cross-Correlation: We use MATLAB’s xcorr function to calculate the cross-correlation between two acoustic signals normalized with respect to power. If the two signals are ‘f’ and ‘g’, the discretized cross-correlation measures the similarity between ‘f’ and shifted (lagged) copies of ‘g’ as a function of the lag. If ‘f’ and ‘g’ have different lengths, the function appends zeros at the end of the shorter vector. [56]
  (4) xcoor(f,g)=(f*g)[lag]
  The function, xcorr, yields different outputs based on the input lag parameter. To calculate the similarity between the two signals, we select the maximum cross-correlation value emitted for any input lag parameter.
  (5) $$\begin{array}{c} xc=\left\{\left(f*g\right)\left[\frac{lag}{lag}\right]\right\}\epsilon \left\{allpossiblelagvalues\right\} \end{array}$$
  (6) $$\begin{array}{c} similarity(x,y)=maximum(xc) \end{array}$$
2. Frequency-Based Distance: The frequency domain distance between a pair of signals is calculated by measuring the Euclidean distance between the frequency spectrum of the two signals. Spectrum is calculated by evaluating the FFT coefficients in the range 0.4–22 kHz [55]. For the two signals ‘*f*’ and ‘*g*’, the frequency-based distance is measured using the following equations.
  (7) $$\begin{array}{c} FFT_{f}=FFT\left(f\right),FFTcoefff\epsilon \left[0.4,22.05\right]kHz \end{array}$$
  (8) $$\begin{array}{c} FFT_{g}=FFT\left(g\right),FFTcoeff\epsilon \left[0.4,22.05\right]kHz \end{array}$$
  (9) $$\begin{array}{c} distance=EuclideanDistance(FFT_{f},FFTs_{g}) \end{array}$$

Time-Frequency analysis is classical approach commonly used in feature extraction in different behavioral authentication mechanisms [42]. In our case, Time-frequency analysis helps in extraction of useful features such as hold time and release time etc. (rest are mentioned in Table 1)) from the acoustic emanations generated from the keystrokes during PIN entry. Based on the features extracted from time-frequency analysis, the system is trained with different machine-learning models. The machine-learning models used are discussed in the next section.

#### 3.2.4. Machine-Learning Models and Assessments

We use the extracted features to train the machine learning models and make predictions. We analyze the robustness against certain criteria by indicating the FAR FRR and TAR of the models.

1. Machine Learning: This work evaluates three machine-learning models, specifically:
  (a) Gaussian Naïve Bayes (Gaussian NB)
  (b) Logistic Regression (LR), and
  (c) Support Vector Machines (SVM)
  In our task, we know the input and we know what the output should be; however, we do not have an algorithm to transform the input to the output. When we meet this situation, we can try machine-learning. Our dataset can be labeled as positive and negative examples, e.g., if the PIN is entered by User 1, we mark it positive, otherwise we mark negative. Supervised learning is suitable for the labeled data [57]. We extract 10 kinds of features from the raw acoustic data, including inter-keystroke latency, hold time, hit peak, release peak, press time, release time, press volume, release volume, press spectrum, release spectrum. We want to find which feature contribute most to help us to make decision. Gaussian Naïve Bayes classifier distributes the same representational power to each feature [58]. Thus, we applied the Gaussian Naïve Bayes classifier to the dataset and found when we use latency and hold time as features, we can get the best result. The latency and hold time are categorical features, the expected outcomes, e.g., User 1 entered the PIN, are categorical as well. In this situation, logistic regression is suited to describe the relationship between features and outcomes [59]. The SVM can add exponents to the feature and raise the accuracy of the model [60], this can neutralize the negative effect of the small number of features in our case. In Section 3.3, our goal is to prove the threat that use acoustic emanation to recover a PIN is possible. Thus, we only trained a logistic regression model. In Section 4, the essential part of this paper, we tried 3 models to reach the best accuracy in user verification.
2. False Acceptance Rate (FAR), False Rejection Rate (FRR) and True Acceptance Rate (TAR): This section provides a description of metrics for the evaluation of Biometric performance. FAR and FRR are used in assessing authentication or verification system, e.g., [61,62]. True acceptance rate means the accuracy of the prediction model. Typically, setting a global acceptance threshold requires considering the tradeoff between the false acceptance rate and false rejection rate.
  (a) FAR: This is the measure of the likelihood that the security system will incorrectly accept an access attempt by an unauthorized user.
    (10) $$FAR=\frac{\mathrm{Number}\;\mathrm{of}\;\mathrm{false}\;\mathrm{acceptance}}{\mathrm{Number}\;\mathrm{of}\;\mathrm{identification}\;\mathrm{attempts}}$$
  (b) FRR: This is the measure of the likelihood that the security system will incorrectly reject an access attempt by an authorized user.
    (11) $$FRR=\frac{\mathrm{Number}\;\mathrm{of}\;\mathrm{false}\;\mathrm{rejections}}{\mathrm{Number}\;\mathrm{of}\;\mathrm{identification}\;\mathrm{attempts}}$$
  (c) TAR: This is the measure of the likelihood that the security system will correctly accept an access attempt by an authorized user.
    (12) $$TAR=\frac{\mathrm{Number}\;\mathrm{of}\;\mathrm{true}\;\mathrm{acceptance}}{\mathrm{Number}\;\mathrm{of}\;\mathrm{identification}\;\mathrm{attempts}}$$

This methodology is devised such that it can be applied and integrated to any POS terminal or machine. The system records the acoustic emanations, then extracts press and release time interval features that are conducive to identify the user for future access attempts. The methodology is non-invasive, meaning that the features are not derived from a data feed generated by the ATM machine, but rather collected externally using a microphone, with background noise cancellation and omission of certain known frequencies like the ATM’s feedback sound. This modular approach significantly reduces the cost of integrating the reinforcement and compares inexpensive to other candidates such as iris detection and fingerprint scanning, as it only requires an additional audio recording module. The methodology presented acts as general framework that is simple to implement and execute on any keyboard or PED keypads. In the following section we shall present our experiment and results in the light of the application-threat model presented above to recover a key PIN.

### 3.3. Experiment and Results

During the PIN entry the microphone collects the acoustic data and performs time frequency decoding [55] to recover the PIN entered by the user. The objective is to analyze the risk of such an attack that can be implemented by shopkeepers on a point of sales terminal (POS) or a self-checkout kiosk. The experiment was performed in the following steps:

- An audio signal repository was established for the key presses 1, 5, 6, 8, 9, and 7 for a targeted user.
- The repository was divided into testing and training sets.
- Then a similarity function was implemented using pairwise cross-correlation.
- Each testing and training keystroke ‘f’, was transformed into a feature vector that consisted of six elements. Each element describes the average similarity of ‘f’ against the acoustic emanations generated from a specific key source. (i.e., feature(f) = similarity to key 1, similarity to key 5, similarity to key 6 and so on)
- The features are then plotted for the press and release regions followed by min-max scaling.

The data analysis considers different inputs and yields outputs that discern the ATM PINs entered by the user.

#### 3.3.1. Time-Frequency Analysis

**Figure 8:**
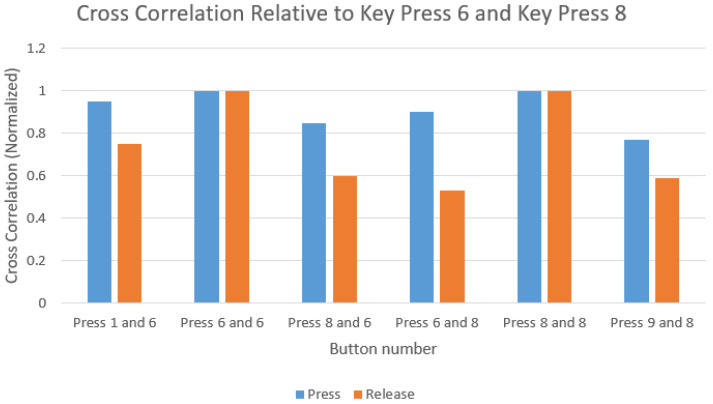
Similarity of press and release of key “6” with key “8”.

**Figure 9:**
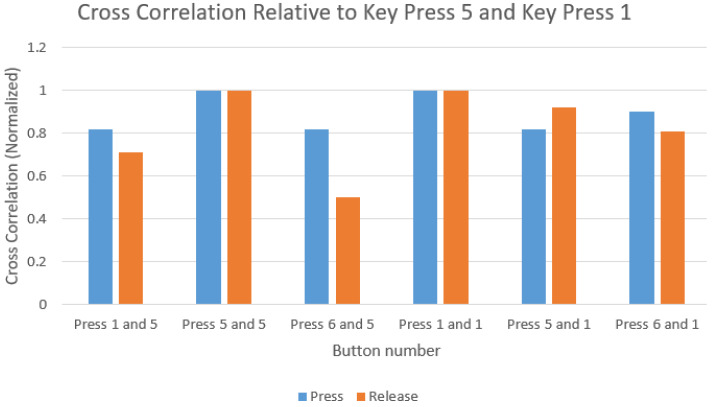
Similarity of press and release of key “5” with key “1”.

Our initial experimental results show that simple cross-correlation analysis of keystroke acoustic emissions could indicate the correct keystroke if trained on a device for a targeted user. The empirical data suggests that emanations produced from a specific key exhibit higher similarity to presses of the same key than that of other keys as shown in Figure 8 and Figure 9.

As the correlation plot itself provided perceptible evidence to discern the source of the keystroke, the implementation of a Logistic Regression model to train and test the data yielded correct predictions for most keystrokes if trained on a targeted user. However, there are a few shortcomings in this approach.

First, we observed a drop in prediction accuracy when the model was trained on multiple users and then tested for a specific user. This indicates that it is not as likely to successfully execute an attack by using dummy users to train the system and then recovering the customer’s PIN by cross-correlating the corresponding key presses. This is because different users employ dissimilar typing styles, which influences the acoustic signal due to varying incident angles and applied pressure. In fact, these differences are due to varying finger positions while entering the PIN and other inherent behavioral characteristics.

Secondly, in the above experiment, the training set also sees other entries with identical PIN sequence for the targeted user. This is a concern because the angle of incidence also depends on the key character pressed immediately before. This influences the signal produced from that particular key button, making it distinct for different PIN sequences. This indicates that it is not so likely to achieve similar results on the customer’s PIN entry if the system is trained to learn on other key sequences like birth date and phone numbers.

Therefore, to compensate for these concerns, the new training set only comprised of PIN sequences that are distinct from the one being tested. This ensures that the single character accuracy remains independent of PIN sequence. Doing so, we can gauge the possibility where the kiosk asks the customer to input his phone number and birth date, to ultimately recover the ATM PIN.

#### 3.3.2. Data Analysis

We consider a Logistic Regression model that takes in a pairwise cross-correlation matrix as the feature inputs, and outputs the predicted keystroke.

(13) $$\begin{array}{c} s_{f,k}:=\left(\frac{\Sigma_{i=1}^{n}similarity\left(f,t\left[i\right]\right)}{n},t=\left\{\mathrm{Acoustic}\;\mathrm{signals}\;\mathrm{generated}\;\mathrm{from}\;\mathrm{key}\;k\right\}\right) \end{array}$$

(14) $$\begin{array}{c} f_{u,i}=\left(S_{f,0},S_{f,1},S_{f,2},S_{f,3},S_{f,4},S_{f,5},S_{f,6},S_{f,7},S_{f,8},S_{f,9}\right) \end{array}$$

(15) $$\begin{array}{c} Input:Similaritiesacquiredthroughtimefrequencydecoding \end{array}$$

(16) $$\begin{array}{c} Output:Key \end{array}$$

**Figure 10:**
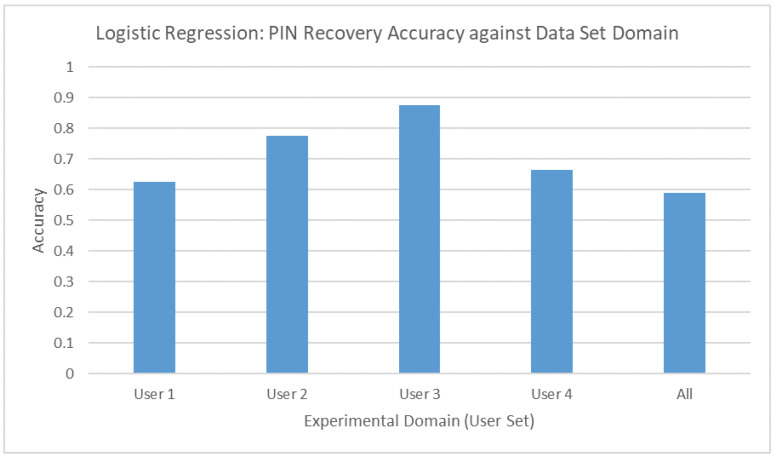
Logistic Regression results for single character detection.

According to the results (Figure 10), it is possible to predict single characters of the PIN sequence to a certain degree of accuracy that lies above 62%. Additionally, the results also indicate that the accuracy when the training domain consisted of various users was not as low as previously expected. This signifies that the cross-correlated features specifically target the button’s mechanical properties instead of behavioral characteristics. Therefore, the threat model presented for this experiment is feasible, provided the customer is duped into revealing a huge set of keystrokes.

## 4. Behavioral Acoustics for Verification of a PIN Key Users

In the last section, we proved that it is possible to infer the PIN key using acoustic data. As a countermeasure, we propose a verification method to defend security from this attack. Our method extends the hypothesis made in [51] that suggests the use of keystroke dynamics to serve as an additional layer in the user authentication workflow. This work proposes that analyzing the way the user types from the acoustic signals emanated during keypress can provide countermeasures against various risks relating to theft and fraudulent impersonation. We shall gauge the reliability of such mechanisms and propose a general architecture to enhance the reliability of authentication systems that impacts on ATM security and workspace controls.

### 4.1. Authentication Based on Acoustic Analytics

**Figure 11:**
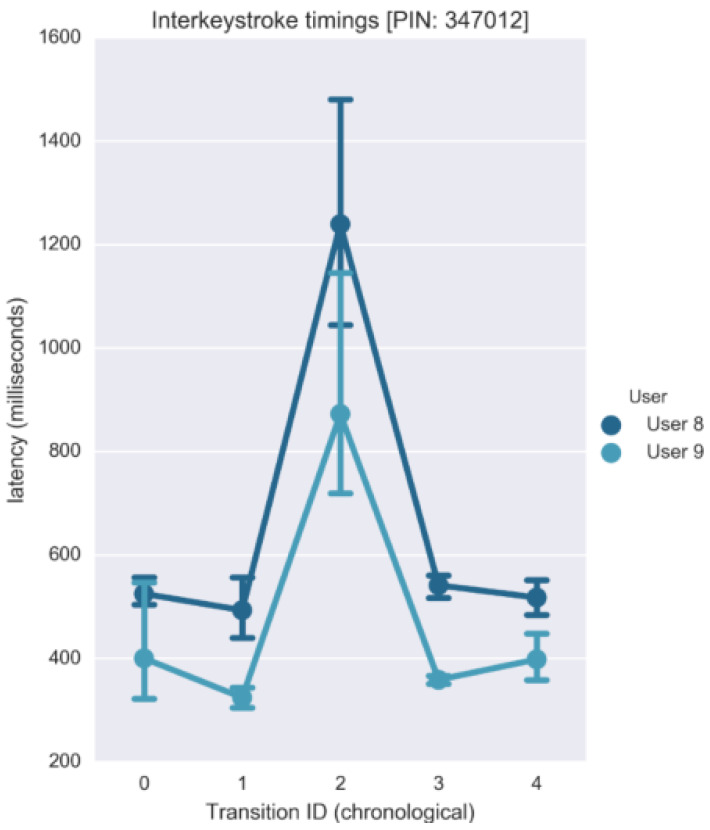
Inter-keystroke variation on 2 users.

The main idea of this study is that the keystroke acoustic data collected over a certain number of times for a particular user, is distinctive enough to construct a cognitive fingerprint for that specific user. Figure 11 shows how the inter-keystroke timings vary between two users for a given PIN sequence (six digits) entered on a PIN Entry device (PED). The vertical lines in the plot indicate the range of latencies exhibited for specific transitions, with the connected dots signifying the mean latencies. For instance, it can be visually inferred from Figure 11 that ‘User 8’ generally takes more time between subsequent keystrokes as compared to ‘User 9’. Features like these are revealing of the user’s psychological signature, which can be used to verify the veracity of new entry attempts.

**Figure 12:**
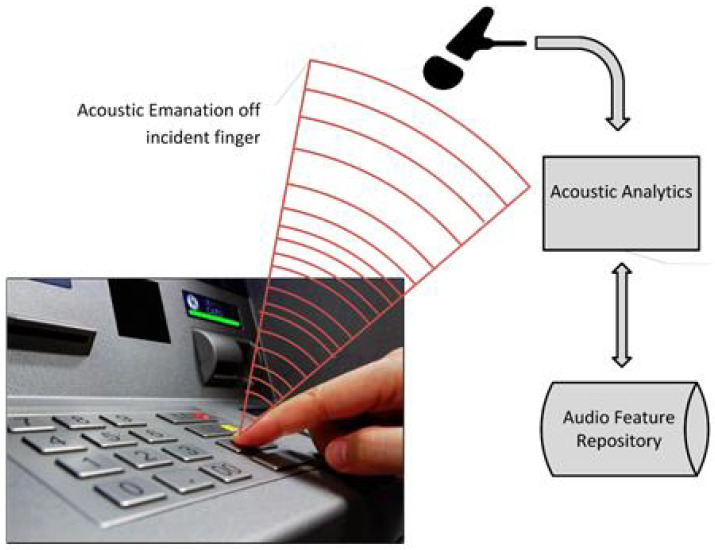
Data collection ATM PED.

The data collected in the previous experiment was obtained from a PIN Entry device (Figure 12) for the PIN sequence ‘347012’. On the keypad, the distance between ‘4’–‘7’ and ‘3’–‘4’ correspond to a ‘short’ and ‘diagonal’ transition, respectively. While it is observed that the transition type (distance) influences the inter-keystroke latency exhibited, these trends are susceptible to pauses that are distinctive to the operating user. At the same time, pauses are beneficial as it is considered by the classification algorithm during user verification.

In the following section, we shall present an application-threat model in which we explain how an adversary can compromise the user specific information, sufficient to authenticate himself as a legitimate user and how authentication based on acoustic analytics is used to create a biometric that can act as an additional layer in the user authentication work flow.

### 4.2. Application-Threat Model: User Authentication Based on Acoustic Analytics of an ATM Machine

Consider the application-threat model where the adversary is assumed to have seized the ATM card and ATM and Personal Identification Number (PIN) of a cardholder using unfair means. The adversary then goes to the ATM machine and authenticates himself successfully as he previously managed to attain the entities that satisfy the ATM’s challenges.

Considering the countermeasures for the above threat model, the only entity that is non-transferable from the victim to the adversary is the biometric characteristics (i.e., typing rhythm, iris recognition). In this case, the third remaining degree is the biometrics to resolve entry-attempt authenticity that acts as a countermeasure to prevent such risks.

#### 4.2.1. Data Analysis and Results

This section focuses on the acoustic analytics performed for the features gleaned from the previous systems. First, we describe the feature set and then detail the models that provide the strongest results.

**Figure 13:**
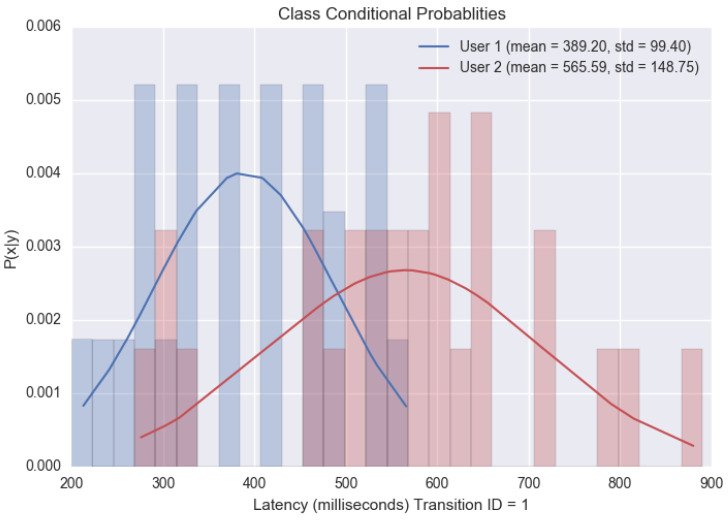
Class-conditioned Probability for two Users typing a specific PIN.

**Figure 14:**
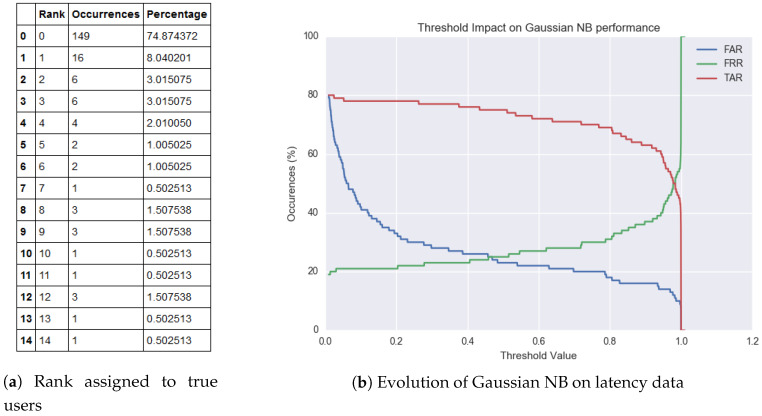
Ranking awarded to true user during Gaussian NB testing.

**Figure 15:**
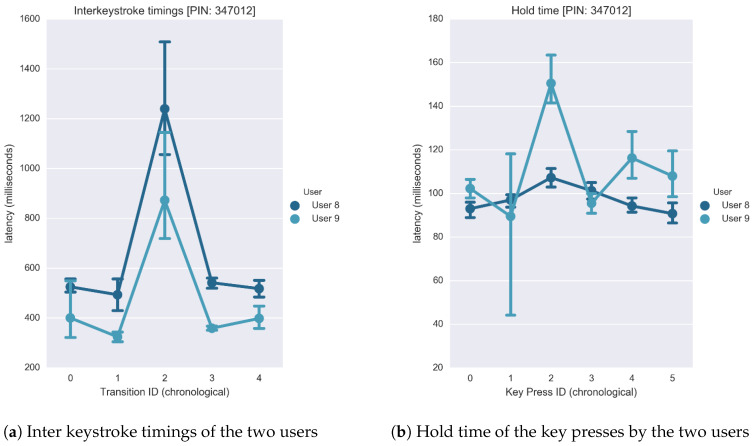
Inter-keystroke Timings and Hold Variation of the two user entries.

**Figure 16:**
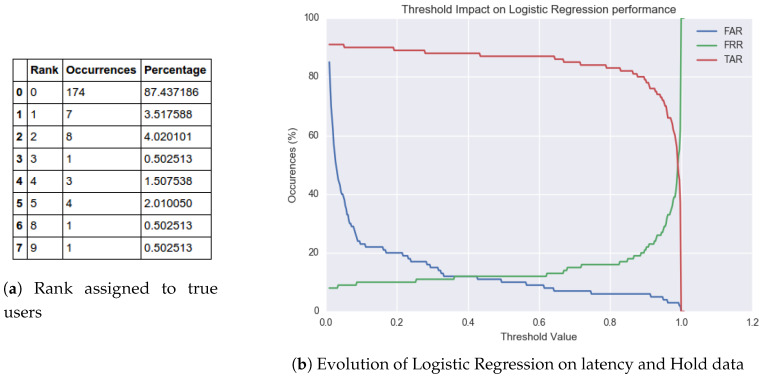
Ranking awarded to true user with Logistic Regression testing.

1. Gaussian Naïve Bayes classifier with Latency features: In this analysis, every PIN sequence is encoded into a feature vector by extracting the latencies associated with all the five transitions in the six-digit numbered PINs. This vector is then fed into a Gaussian Naïve Bayes classifier that trains itself to output the predicted users.
  (17) $$\begin{array}{c} l_{u,i,j}=\mathrm{latency}\;\mathrm{for}\;transition_{j}\;\mathrm{in}\;\mathrm{the}\;\mathrm{PIN}\;\mathrm{entry}\;sample_{i}\;\mathrm{collected}\;\mathrm{from}\;\mathrm{User}\;u \end{array}$$
  (18) $$\begin{array}{c} f_{u,i}=\left(l_{u,i,1},l_{u,i,2},l_{u,i,3},l_{u,i,4},l_{u,i,5}\right) \end{array}$$
  (19) $$\begin{array}{c} Input=\{Latencies\} \end{array}$$
  (20) $$\begin{array}{c} Output=User \end{array}$$
  Figure 13 shows the class-conditioned probabilities of observing a particular latency feature ‘x’ for a given class ‘y’ for the first transition of a given PIN sequence. Likewise, the model trains itself for all five transition to discern various users in the training set. Once trained, we analyze the accuracy of this model on our testing set. Again, the test set consists of latency-based features with the vector cardinality being five. For every test instance, the model outputs a probability vector, which depicts the model’s confidence for every user. We derive a ranking order from the probability list. According to the formulation, rank ‘0’ is assigned to the user that the model believes is the correct answer, rank ’1’ is the second strongest prediction, and henceforth. The table in Figure 14 depicts the rankings awarded to the actual users during the testing stage. A model is considered good if the occurrences where it rewards the true user a rank ‘0’ is high. To better understand the predictions, we plot the False Acceptance Rate (FAR), False Rejection Rate (FRR) and True Acceptance Rate (TAR) yielded for various confidence threshold values.
2. Gaussian Naïve Bayes classifier with Latency and Hold features: Here, we consider the case where the feature vector consists of latency and hold-based features. We retain the feature set collected from the previous section and concatenated it with the features acquired for the hold time. The hold time for a keystroke is evaluated by measuring the latency between the press and release starting points. Figure 15 provides a visual representation of the many non-overlapping features that assist the classifier to discriminate users.
  (21) $$\begin{array}{c} h_{u,i,j}=\mathrm{hold}\;\mathrm{time}\;\mathrm{for}\;keypress_{j}\;\mathrm{in}\;\mathrm{the}\;\mathrm{PIN}\;\mathrm{entry}\;sample_{i}\;\mathrm{collected}\;\mathrm{from}\;\mathrm{User}\;u \end{array}$$
  (22) $$\begin{array}{c} f_{u,i}=\left(l_{u,i,1},l_{u,i,2},l_{u,i,3},l_{u,i,4},l_{u,i,5},l_{u,i,6}\right) \end{array}$$
  (23) $$\begin{array}{c} Input=\{Latency+Hold\} \end{array}$$
  (24) $$\begin{array}{c} Output=User \end{array}$$
  From Figure 15, it is evident by the increase in number of true users labeled ‘0’ that the classifier’s performance has incremented with the inclusion of hold time. This corroborates the claim that the ‘hold time’ exhibits user specific characteristics. Furthermore, it can be inferred from the figure that a confidence threshold value at around 80% (0.8), can yield an approximate 80% TAR, 20% FAR, and 20% FRR. In addition to that out of all the 37 instances that were incorrectly not ranked ‘0’, there was only one occurrence of two consecutive false rejections. This means that if a user is falsely rejected by an authentication system, then the likelihood of being rejected again in the next attempt is negligible.
3. Logistic Regression with latency and hold features: In this segment, we consider a change in the machine-learning model as compared to the previous section of the paper. The generative Gaussian Naïve Bayes model is replaced with a discriminative Logistic Regression model, and the input only consists of latency and hold-based features.
  (25) $$\begin{array}{c} Input:\{Latency+Hold\} \end{array}$$
  (26) $$\begin{array}{c} Output:User \end{array}$$
  Compared to the previous techniques, Figure 16 shows an increase in performance. In fact, this model achieved the highest accuracy for this experiment. The number of actual users awarded the rank ‘0’ increased from 80% to 87%. Similarly, setting the threshold at a confidence value of 40% (0.4) achieved an 88% TAR, 12% FAR, and 12% FRR. Additionally, there was no such instance recorded where there were two consecutive false rejections. Since this data set only consists of 15 users, the occurrences of false rejection will be higher when rolled out for a vast user domain. Although rare, as we consider entry attempts by all false users and train on a small data set, it is important to counteract this scenario by letting the user type in the PIN sequence again. The likelihood of the user being rejected in the second attempt is low, and the user is challenged up to three times before complete access denial. However, on the downside, allowing three attempts could also have a detrimental impact on the false acceptance rate.
4. Support Vector Machine with Latency and Hold: In this section, we gauge the performance of a Support Vector Machine model to classify the data.
  (27) $$\begin{array}{c} Input:\{Latency+Hold\} \end{array}$$
  (28) $$\begin{array}{c} Output:User \end{array}$$
  The performance was found to be similar to Gaussian Naïve Bayes, but less accurate compared to logistic regression, Table 2 shows the accuracy of SVM.

#### 4.2.2. Enhanced Authentication Architecture

**Figure 17:**
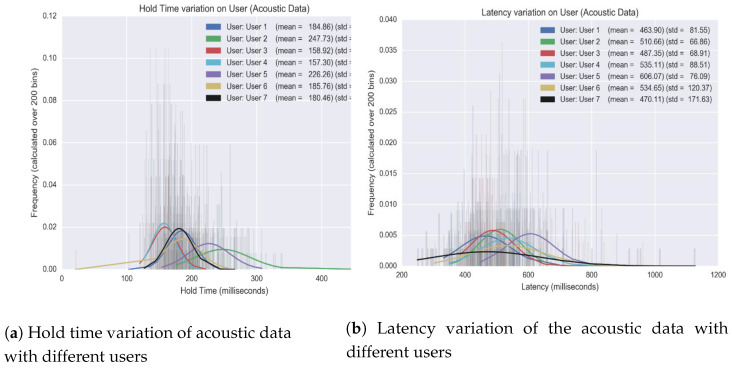
Hold and Latency variation with different users.

We investigated several machine-learning models and feature engineering techniques that could serve as a behavioral biometric for user verification during PIN entry. The overall results of these models are shown in Table 3. The first column of the Table 3 shows the machine-learning models that we used; the second column shows the features that we used in the machine-learning model; the third to fifth column show the false acceptance rate, false rejection rate and true acceptance rate respectively. The detail explanation of FAR, FRR and TAR can be found in Section 3.2.4. We started with simplest method that is by using Gaussian NB model with a single feature that is latency, the results in FAR to 22%, FRR to be 22% and TAR to be 75%. This mean that the 75% of the time, system is able to correctly identify the authorized user attempt for PIN entry (i.e., TAR) whereas, 22%–24% of the time either the system incorrectly accepts an access attempt by an authorized user or the system incorrectly rejects an access attempt by an authorized user. Similarly, are the rest of the results shown in Table 3. Importantly, we deduce that Logistic Regression as a means to train on latency and hold-based features is sufficient (results in TAR to be 88%, the best compared to the result models used) to verify user authenticity. Figure 17 shows the variation of hold time and latency of different users. The vertical axis of Figure 17 means the occurrence rates of hold time and latency features; the horizontal axis means the numbers of hold time and latency, respectively. The histograms in Figure 17 show the occurrence rate of each hold time or latency of different users; the curves show the general distribution of hold time or latency of different users, respectively. The proposed fool proof extendable model provides a cost effective, backward compatible, resilient biometric verification system that improves classifications and adapts to behavioral rhythms over time. As the training set is relatively small, it exemplifies the quick learning rate during the enrolment phase.

The experiment was carried out by requesting the users to select a PIN from a list of five PIN sequences and completing the training and testing procedures that follow. Once completed, the user moves to the next PIN sequence in the list and repeat the exercise. In this survey, each PIN sequences (four digits) is entered 11 times as a part of the training phase and another ten times as testing. Subjects also maintained a consistent typing style throughout the exercise.

## 5. Conclusions

In this paper, we addressed three practical scenarios that are influenced by the emergence of keystroke dynamics, side-channel analysis, and machine learning. This research evaluated the feasibility of these scenarios in contexts that have never been explored before and examines various frameworks. First, we devised a PIN key recovery attack and used the acoustic signal to successfully recover 4–6-digit random PINs from the emanations generated from the keystrokes. Secondly, we proved that it is possible to verify user identity from acoustic emanations. Based on the results, we proposed a defense mechanism to thwart user impersonation attempts and several other risks, thus elevating the security of the PEDs. We plan to improve our experiment in the future. A limitation of our work is the size of training set. For a PIN, a user only enters 4 times in our experiment. We consider increase the number to 8 or 10 times per PIN. Extension of this work is in the plan as well. As we already proved that time interval between keystrokes can be used to verify user, we suggest this technique can be applied in other area. Also, different (supervised) machine-learning models are also be tested. One possible application is remote authentication. When user enters password on the website, the time intervals between keystrokes are recorded and used to verify the user’s identity. Another potential application is mobile phone unlocking. Due to the impact of Covoid-19, iPhone users must wear masks and cannot use face ID (a technique that can detect user’s face to verify the user) to unlock their phone. Instead they must use PIN, which is possible to be record by others. We plan to record the time intervals between two presses on screen and use these to verify user.

## Figures and Tables

**Table 1:** Compelling features observed in acoustic signals.

Feature	Unit
Inter-Keystroke latency	Time
Hold time	Time duration
Hit Peak	Time
Release Peak	Time
Press Time	Time duration
Release Time	Time duration
Press Volume	Volume against Time
Release Volume	Volume against Time
Press Spectrum	FFT coefficients
Release Spectrum	FFT coefficients

**Table 2:** Rank Assigned to the True Users (SVM).

Rank	Occurrences	Percentage
0	163	81.9096
1	21	10.5528
2	7	3.5179
3	1	0.5025
4	2	1.0050
5	1	0.5025
6	1	0.5025
7	1	0.5025
8	2	0.0050

**Table 3:** Overall results (Acoustic Biometric Verification).

Model	Feature	FAR	FRR	TAR
Gaussian NB	Latency	22%	22%	75%
Gaussian NB	Latency and Hold	20%	20%	81%
Logistic Regression	Latency and Hold	12%	12%	88%
Support Vector Machines	Latency and Hold	18	18	82%
